# Characterisation of proguanylin expressing cells in the intestine – evidence for constitutive luminal secretion

**DOI:** 10.1038/s41598-019-52049-0

**Published:** 2019-10-30

**Authors:** Florent Serge Dye, Pierre Larraufie, Richard Kay, Tamana Darwish, Juraj Rievaj, Deborah A. Goldspink, Claire L. Meek, Stephen J. Middleton, Richard H. Hardwick, Geoffrey P. Roberts, Jennifer L. Percival-Alwyn, Tris Vaughan, Franco Ferraro, Benjamin G. Challis, Stephen O’Rahilly, Maria Groves, Fiona M. Gribble, Frank Reimann

**Affiliations:** 1 Wellcome/MRC Institute of Metabolic Science, University of Cambridge, Cambridge, UK; 2 Department of Antibody Discovery and Protein Engineering, R&D, AstraZeneca, Cambridge, UK; 3 Dosage Form Design & Development, AstraZeneca, Cambridge, UK; 4 Department of Gastroenterology, Cambridge University Hospitals NHS Foundation Trust, Cambridge, UK; 5 Barrett’s Oesophagus and Oesophago-gastric Cancer, Gastroenterology Services, Addenbrooke’s Hospital, Cambridge, UK; 6 Research and Early Development, Cardiovascular, Renal and Metabolism, BioPharmaceuticals R&D, AstraZeneca, Cambridge, UK

**Keywords:** Gastrointestinal hormones, Preclinical research

## Abstract

Guanylin, a peptide implicated in regulation of intestinal fluid secretion, is expressed in the mucosa, but the exact cellular origin remains controversial. In a new transgenic mouse model fluorescent reporter protein expression driven by the proguanylin promoter was observed throughout the small intestine and colon in goblet and Paneth(-like) cells and, except in duodenum, in mature enterocytes. In Ussing chamber experiments employing both human and mouse intestinal tissue, proguanylin was released predominantly in the luminal direction. Measurements of proguanylin expression and secretion in cell lines and organoids indicated that secretion is largely constitutive and requires ER to Golgi transport but was not acutely regulated by salt or other stimuli. Using a newly-developed proguanylin assay, we found plasma levels to be raised in humans after total gastrectomy or intestinal transplantation, but largely unresponsive to nutrient ingestion. By LC-MS/MS we identified processed forms in tissue and luminal extracts, but in plasma we only detected full-length proguanylin. Our transgenic approach provides information about the cellular origins of proguanylin, complementing previous immunohistochemical and *in-situ* hybridisation results. The identification of processed forms of proguanylin in the intestinal lumen but not in plasma supports the notion that the primary site of action is the gut itself.

## Introduction

Guanylin is a small peptide produced in the intestinal mucosa from the *Guca2a* gene which has established roles in intestinal fluid homeostasis and maintenance of gut physiology. Together with the related peptide uroguanylin (encoded by the *Guca2b* gene), and heat-stable enterotoxin STa, guanylin activates the Guanylate Cyclase C receptor (GC-C)^1^, which is encoded by the *Gucy2c*-gene and expressed along the gastrointestinal (GI) tract. Unusually for a gut peptide/receptor combination, however, the peptide appears to access its receptor from the luminal direction^2–4^. GC-C activation catalyses the generation of cGMP, stimulating transepithelial chloride flux, inhibition of sodium proton exchangers^5^, and increase in water movement into the intestinal lumen^6^. Overstimulation or gain of function mutations in GC-C lead to altered GI transit diarrhoea, and loss of function mutations have been associated with meconium ileus in infants^7–9^.

The reported active 15 amino acid bioactive peptide guanylin derives from the C-terminus of a longer pre-proguanylin peptide, which comprises a signal sequence (residues 1–21) and proguanylin (residues 22–116 in the mouse sequence, 22–115 in the human). A number of reports have suggested that proguanylin is the predominant secreted form of the peptide, but the enzymatic pathway underlying proguanylin processing to generate bioactive guanylin remains unclear^10^. Previous studies aimed to address the potential cellular origin of guanylin. Early reports suggested, based on the hormonal function proposed for guanylin peptides, that enteroendocrine cells were the likely source of these peptides, including enterochromaffin cells^11,12^ and somatostatin secreting D-cells^13^. However, other cell types were also reported as sources of guanylin, including goblet cells^14–16^, Paneth cells^10,17^, colonic Paneth-like cells^16^, tuft cells and enterocytes^16,18^, but with some discrepancies between techniques and studies. Stimuli underlying proguanylin secretion remain poorly characterised, with the strongest body of evidence supporting a link to salt consumption^19,20^, corresponding with an observed increase in *Guca2a* expression in the intestines of rats fed a high salt diet^21^.

The GC-C signalling axis has been reported to play a role in crypt-villus epithelial proliferation and to act as a tumour suppressor gene. Reduced GC-C receptor signalling was linked to hyperplasia of crypts and villi along the gastrointestinal tract and was associated with increased susceptibility to tumorigenesis^22^. In a cohort of patients with stage I-III colorectal cancer, guanylin mRNA and peptides were lost and/or significantly lower in cancerous tissues compared to healthy adjacent tissues in >85% of cases^23^, and targeting the GC-C pathway at the early stages of colorectal cancer has been proposed as a candidate therapeutic strategy^24–27^.

Circulating proguanylin levels were lower in people with obesity and raised following Roux-en-Y gastric bypass surgery^28^, suggesting potential links to metabolism or food intake. Corresponding with these findings, mice fed a high fat diet had lower expression and peptide levels of guanylin and forced re-expression of guanylin reduced the rate of obesity associated colorectal cancer^29^. Gucy2c-deficient mice are hyperphagic and heavier compared with wild-type mice^30^. In wild-type mice, food intake was reduced following intravenous administration of prouroguanylin, but not proguanylin, and increased following treatment with a prouroguanylin antiserum and it was speculated local processing in the hypothalamus releases active uroguanylin^30,31^, suggesting a central role for GC-C signalling. However, another group found that neither systemic nor central administration of proguanylin-derived peptides modulated food ingestion or glucose homeostasis in mice^28^, despite targeting the same GC-C receptor. GC-C activation has, however, been shown to stimulate secretion of the anorectic peptide glucagon-like peptide-1 (GLP-1) from enteroendocrine cells in the GI tract^32^.

Despite the multiple proposed physiological roles of guanylin peptides and increased interest in their use for treating irritable bowel syndrome or colorectal cancer^33^, the cellular origins of guanylin and the mechanisms underlying its secretion are poorly understood. To address these questions, we generated a transgenic mouse model in which the *Guca2a* promoter drives the expression of the yellow fluorescent protein Venus. This model was utilised alongside the use of mass spectrometry and a newly-established monoclonal antibody-based immunoassay to measure proguanylin and proguanylin-derived peptides in human plasma, tissues and cell supernatants isolated from preclinical experimental systems.

## Results

### Proguanylin levels in human plasma

**Figure 1:**
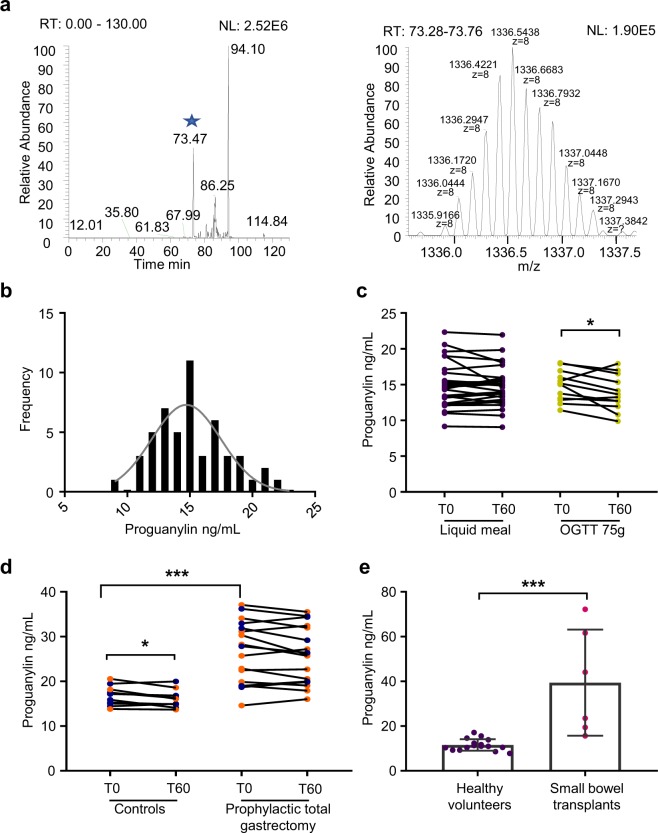
Proguanylin detection in plasma. (**a**) LC-MS/MS evidence of full length proguanylin in human plasma. Right panel shows the total ion chromatogram for an m/z range corresponding to the proguanylin in a charge state of 8 (±0.01 around each isotopic variants), asterisk marks the peak corresponding to proguanlyin, left panel is the m/z distribution at retention time corresponding to the peak marked by an asterisk. (**b**) Data distribution and normality for proguanylin concentration in plasma of fasted healthy volunteers (n = 51). Skewness = 0.37; kurtosis = −0.08; P = 0.69 when evaluated for Shapiro-Wilk test. (**c**) Effect of liquid meal (n = 28) or 75 g OGTT (n = 12) on plasma proguanylin levels in healthy volunteers after 60 minutes. (**d**) Effect of 50 g glucose ingestion on plasma proguanylin levels of healthy controls and participants with total gastrectomy after 60 minutes (n = 17, controls: n = 11). Patients with a mutation in the *CDH1* are indicated in orange. (**e**) Serum levels of proguanylin in fasting healthy volunteers and patients following intestinal transplantation. *p < 0.05 and ***p < 0.001 using a paired t-test when comparing intra individual values across two time points or unpaired t-tests when comparing two groups of individuals.

By LC-MS/MS, we detected high levels of proguanylin (22–115) in human plasma, but were not able to detect shorter proguanylin-derived peptides containing the C-terminal active sequence (Fig. 1a). Therefore, this suggests that proguanylin is mostly circulating as the intact profom which we could detect using a newly developed proguanylin immunoassay. Fasting plasma proguanylin levels in healthy humans were 15.2 ± 2.7 ng/mL (mean ± sd), and followed a normal distribution (Fig. 1b). Plasma levels fell slightly after a 75 g or 50 g oral glucose tolerance test but there was no significant effect of a mixed liquid meal (Fig. 1c,d). Lean patients who had undergone total gastrectomy with Roux-en-Y reconstruction for gastric cancer had elevated fasting proguanylin concentrations (26.5 ± 6.9 ng/mL)), but no significant change after oral glucose ingestion (Fig. 1d). Although a number of the gastrectomy patients carried risk alleles in *CDH1*, neither pre- nor post-operative proguanylin levels appeared influenced by the *CDH1* genotype. Fasting proguanylin concentrations were elevated in a proportion of subjects who had undergone small bowel transplantation (Fig. 1e). One transplant subject had blood samples collected after removal of the small and large intestine and again after bowel transplantation, revealing very low proguanylin after intestinal removal (~0.2 ng/ml, close to the detection limit of the assay) but a normal level (19.4 ng/ml) after transplantation. Collectively, these results provide strong support for the idea that the gut is the predominant source of proguanylin in human blood, which circulates at relatively high levels compared to other intestinally derived hormones, such as GLP-1.

### Identification of proguanylin-derived peptides by LC-MS/MS

**Figure 2:**
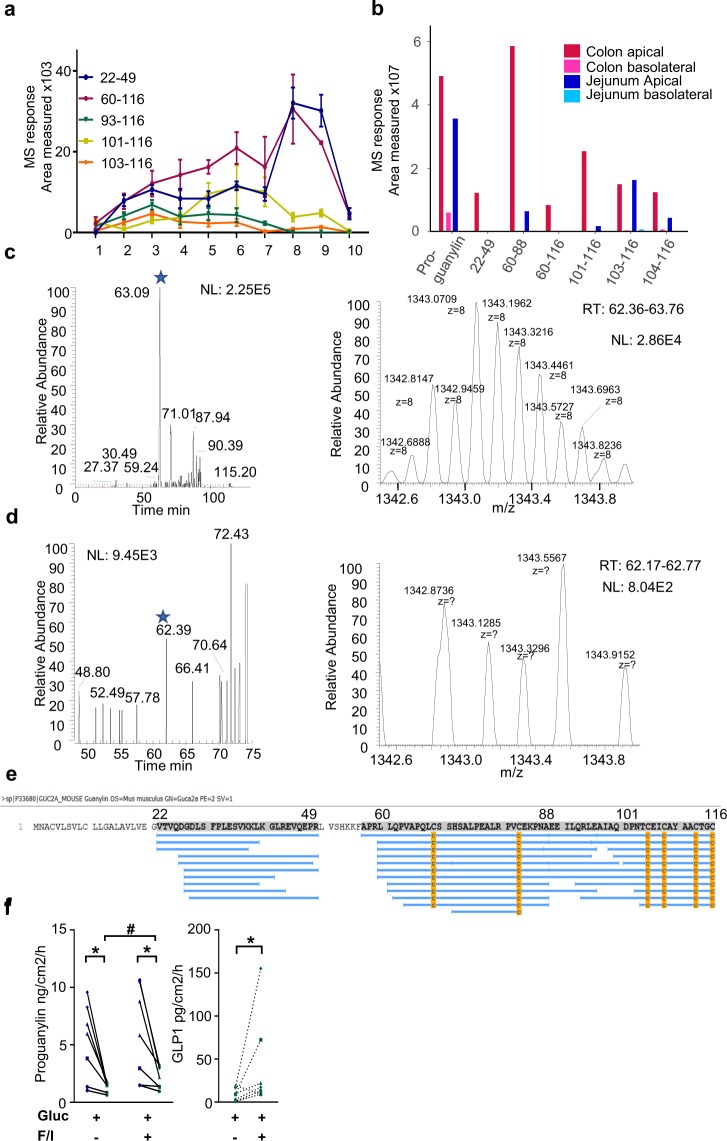
Proguanylin production and secretion in the gut. (**a**) Measurement of GUCA2A peptides along the mouse intestine from duodenum to rectum (1 to 7 are small intestine regions every 5 cm from duodenum to ileum, and 8 to 10 are proximal colon, distal colon and rectum respectively) analysed by LC-MS/MS. Data are mean ± SEM, n = 4. (**b**) Apical (dark colours) and basolateral (light colours) secretion of guanylin peptides from mouse colonic (red) and jejunum (blue) tissues). (**c**,**d**) Evidence of full length proguanylin (marked by asterisk) in mouse jejunum apical compartment (**c**) and absence in the basolateral compartment (**d**) showing chromatogram for the m/z range corresponding to the [M + 8 H]^8+^ charge state of proguanylin (left panel) and the m/z distribution at the expected retention time. (**e**) PEAKS peptidomics search results showing peptides (of less than 65 amino acids) matching the proguanylin sequence in the apical side of the Ussing chamber experiment using murine colonic tissue. (**f**) Apical (blue) and basolateral (green) secretion of proguanylin and basolateral secretion of GLP1 from human colonic tissues using Ussing chambers from 6 tissue preparations from 4 different donors. Data are represented as mean ± SD * and ^#^p < 0.05 using a paired t-test adjusting for multiple testing, comparing apical vs basolateral secretion and unstimulated vs F/I stimulated.

Analysis of tissue homogenates from sequential sections along the mouse small intestine, colon and rectum^34^ by LC-MS/MS did not detect proguanylin itself, likely because other peptides eluted with similar retention times, but identified several proguanylin-derived peptides (Fig. 2a). Whilst these were barely detectable in homogenates from the proximal duodenum, levels increased along the GI tract before falling again in the rectum. Interestingly, the canonical sequence for the reported active 15 amino acid peptide (position m-102–116) was present at extremely low levels in tissue homogenates and detectable only in jejunum and colon, whereas a form containing one additional amino acid at the N-terminus was readily detected in all samples.

Similar analysis of the apical and basolateral solutions from murine jejunum and colon mounted in Ussing chambers (after 120 min each from one colon and one jejunum) identified full length proguanylin (22–116) in both solutions, although at much higher concentrations in the apical compartment (Fig. 2b–d). In the apical compartments of both tissues we also identified several proguanylin derived peptides such as 60–88, 101–116, 103–116 and 104–116 (Fig. 2b,e). The same peptides were detected in the basolateral compartments, but at much lower concentrations, mirroring the lower levels of proguanylin. Immunoassay measurements of proguanylin from human colonic tissues mounted in Ussing chambers also revealed much higher levels released into apical than basolateral compartments (Fig. 2f). Forskolin + IBMX had only a minor effect on proguanylin secretion, although this was a strong stimulus of GLP-1 release into the basolateral compartment, measured in parallel.

### Mapping guanylin-expressing cells along the murine gut axis

**Figure 3:**
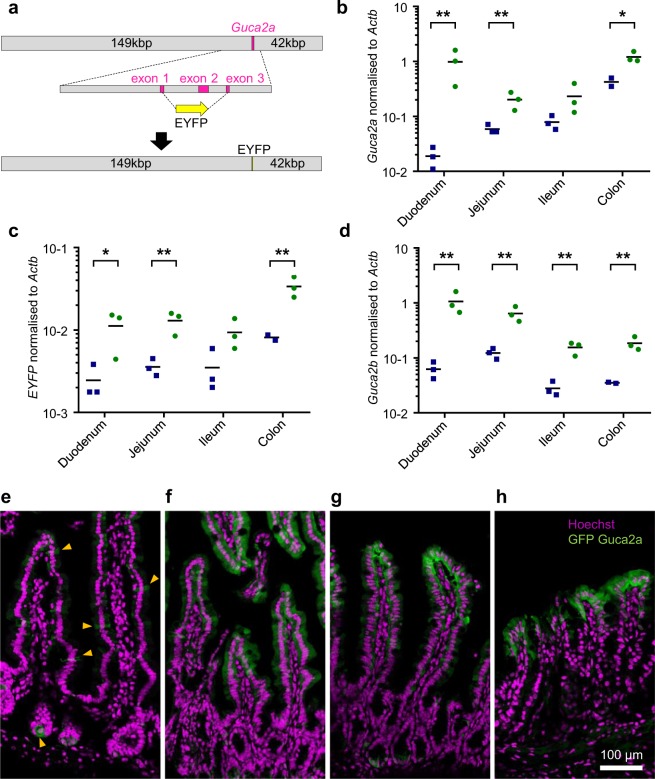
Generation and characterisation of the guanylin-Venus mouse. (**a**) Schematic depicting the insert of BAC RP23-104G3 and replacement of the *Guca2a* coding region from exons 1–3 with the yellow fluorescent protein Venus. Lengths (in kb) of the murine genomic sequence 3′ and 5′ of the *Guca2a* coding region in the BAC are indicated. (**b**–**d**) RT-qPCR analysis of FACS sorted positive (green) and negative (blue) populations for each tissue for *Guca2a* (**b**), *Venus* (**c**) and *Guca2b* (**d**) n = 3 each; negative and positive populations were compared using paired t-test *P < 0.05 **P < 0.01. (**e**–**h**) duodenum (**e**), jejunum (**f**), ileum (**g**) and colon (**h**) of Guca2a-Venus mice immunostained for GFP (green) and DNA (Hoechst; purple).

We were unable to detect specific immunohistochemical staining with the monoclonal antibodies isolated during the development of the new immunoassay in human intestinal tissue slices or primary mixed epithelial cultures (data not shown). Instead, a transgenic mouse model was designed to identify which cell types produce proguanylin, by expression of the fluorescent protein Venus under the control of the guanylin promoter. The line was derived from one founder in which a BAC construct containing the Venus sequence in place of the coding sequence of *Guca2a* was incorporated into the mouse genome (Fig. 3a). Offspring showed fluorescence in the small and large intestine, enabling the use of fluorescence-assisted cell sorting (FACS) to separate fluorescent (positive) from non-fluorescent (negative) cells separately from the duodenum, jejunum, ileum and colon. RT-qPCR analysis of sorted positive and negative cell populations showed enrichment of *Guca2a* and *Venus* expression in the fluorescent populations, thus validating the model (Fig. 3b,c). Interestingly, the fluorescent cell populations were also enriched for the expression of uroguanylin, *Guca2b* (Fig. 3d).

The proportion of Venus fluorescent cells was highly variable between different intestinal tissues. Using flow cytometry to count the number of cells exhibiting fluorescence above a fixed threshold, we estimated that the Venus-positive cell frequency was ~3% of the epithelial cell population in the duodenum, ~50% in the jejunum, 30% in the ileum and 20% in the colon. Immunostaining of tissue sections confirmed this disparity in guanylin-expressing cell distribution along the intestinal axis (Fig. 3e–h). Duodenum contained only scarce Venus positive cells that were scattered along the crypt-villus axis (Fig. 3e). In jejunum and ileum, Venus positive cells appeared to separate into two distinct populations, one located at the tops of the villi and another in the crypts. In colon, the fluorescent marker was largely restricted to the surface epithelium at the top of the crypts (Fig. 2h). These staining patterns suggest that *Guca2a* is expressed in mature enterocytes in the jejunum, ileum and colon but not duodenum, as well as in some other epithelial cell types.

### Identification of guanylin-expressing cells by transcriptomic profiling

**Figure 4:**
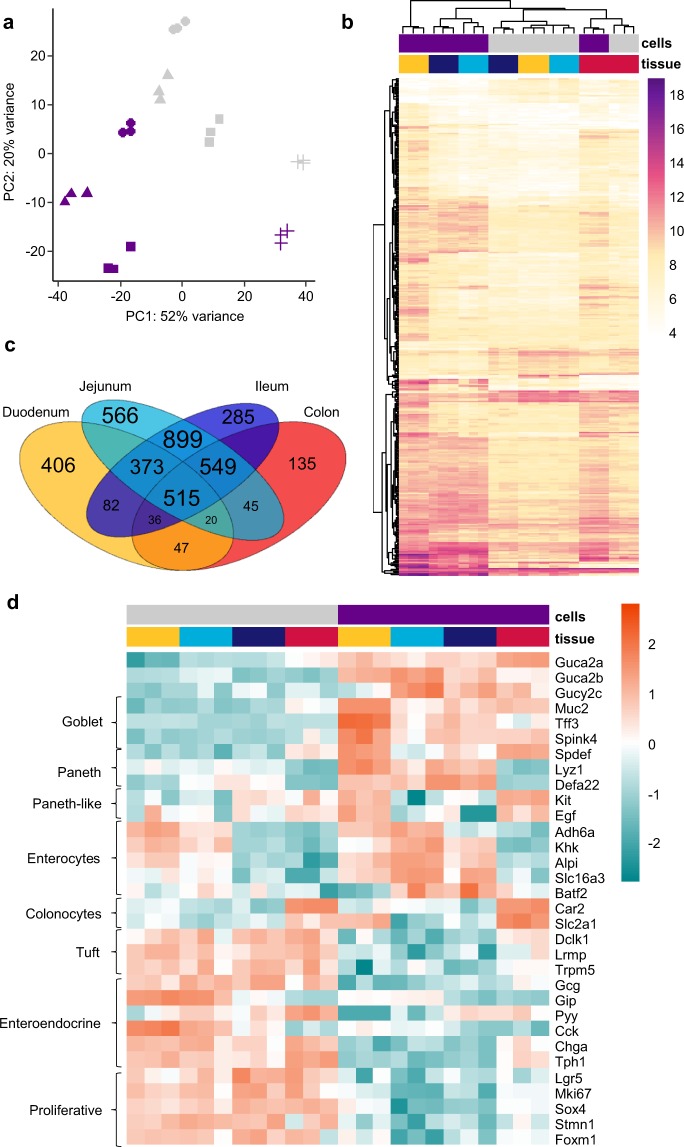
Transcriptome analysis of Guca2a-expressing cells. (**a**) Principal-Component analysis of the top 500 most variable genes between positive and negative cells from the four regions of the GI tract. Non-coding and mitochondrial genes were excluded. In purple are the Venus-positive cell subpopulations and in grey are the negative population. Tissue of origin is represented by shape (circle: duodenum; triangle: jejunum; square: ileum; cross: colon). (**b**) Heatmap showing the top 500 most differentially expressed genes between positive and negative cells across all 4 regions. Values are log10 normalised read counts. Genes and samples are grouped via hierarchical clustering based on Euclidean distance and complete linkage. Colon = red; duodenum = yellow; jejunum = light blue; ileum = dark blue. Purple = positive cells; grey = negative cells (**c**) Venn diagram summarising the overlap of positively-enriched genes in the guanylin-positive cell subpopulation for each region. Positively enriched genes were selected with p-adjusted < 0.05. (**d**) Heatmap of selected cell markers (rows) between positive and negative cells (columns sorted by cell type and tissue of origin). Values are log fold change compared to mean expression across all samples. Colour coding as for (**b**).

To determine the identity of the fluorescent cells in the small and large intestine of Guanylin-Venus mice, we performed RNA sequencing analysis of FACS-sorted Venus-positive and -negative cell populations from four different regions of the GI tract of three mice each. Confirming the RT-qPCR data, both *Guca2a* and *Guca2b* were enriched in fluorescent cell populations from all four intestinal regions, and surprisingly, we also detected higher expression of the receptor GC-C (Gucy2c) in fluorescent cells from the jejunum, ileum and colon (Fig. 4d), although not duodenum. By principal component analysis (PCA) of the RNAseq results, the samples were found to cluster both by the tissue of origin and by whether they were fluorescent or non-fluorescent, indicating that *Guca2a* expressing cells are transcriptomically different from negative cells, and that they vary along the GI tract (Fig. 4a). Analysis of the top 500 most differentially expressed genes across all groups revealed clear separations between *Guca2a* positive and negative cells, and between *Guca2a*-expressing cells in the duodenum compared with their counterparts in the jejunum and ileum (Fig. 4b). Analysis of the numbers of *Guca2a*-cell enriched genes in different intestinal regions also revealed strong similarity between *Guca2a*-positive cells from the ileum and jejunum compared with those from the duodenum (Fig. 4c). 515 genes were enriched in *Guca2a*-positive cells compared to negative cells from the same region, in all four tissues, suggesting at least a degree of overlap between the *Guca2a* cell populations from different regions of the GI tract.

To identify the cell types expressing *Guca2a*, we examined the expression of known marker genes for different epithelial cell populations: goblet cells, enterocytes, Paneth cells, tuft cells, enteroendocrine cells and proliferative cells (Fig. 4d and Supplementary Fig. 1). Goblet cell markers (*Muc2*, *Tff3*, *Spink4*) were enriched in positive cells across all four tissues. Enterocyte cell markers (*Adh6a*, *Khk*, *Alpi*, *Slc16a3*, *Batf2*) were enriched in guanylin-expressing cells, particularly in the jejunum and ileum, and colonocyte cell markers (*Car2*, *Slc2a1*) were also enriched in guanylin-expressing cells. Paneth cell markers (*Lyz1* and *Defa22*) were enriched in positive cells from the small intestine but not colon, consistent with the fact that Paneth cells are not present in the colon, but Paneth-like cells markers (*Kit*, *Egf*) were enriched in positive cells of the colon^35^. Markers of Tuft cells (*Dclk1*, *Lrmp*, *Trpm5*), enteroendocrine cells (including *Gcg*, *Gip*, *Pyy*, *Cck*, *Chga*, *Tph1*) and proliferative cells (*Lgr5*, *Mki67*, *Sox4*, *Stmn1*, *Foxm1*) were relatively depleted in *Guca2a*-positive compared with negative cells.

Overall, this transcriptomic analysis suggests that *Guca2a* is expressed by several different cell populations in the intestinal epithelium: goblet and Paneth/Paneth-like cells in all 4 intestinal tissues, and enterocytes/colonocytes in tissues distal to the duodenum. Notably, *Guca2a* labelled cells did not exhibit expression patterns typical of enterocytes in the duodenum, or of enteroendocrine cells, tuft cells or proliferative cells.

### Identification of guanylin-expressing cells by immunostaining

**Figure 5:**
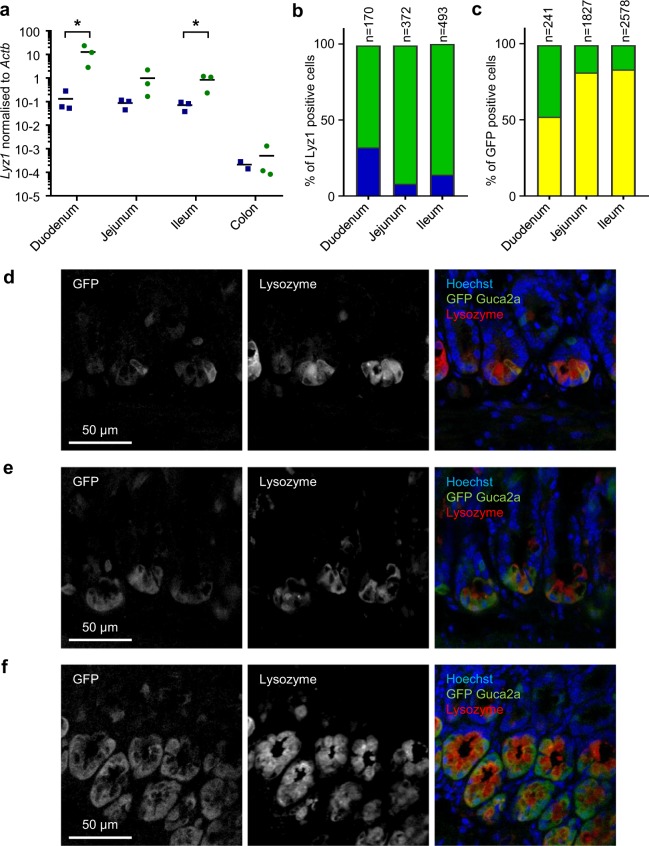
Guanylin expression in Paneth cells. (**a**) Venus-positive (green) and negative (blue) subpopulations of isolated intestinal cells from the Guca2a-Venus mice were analysed by RT-qPCR for *Lyz1* expression. n = 2–3 each; positive and negative populations were compared using a paired t-test *P < 0.05. (**b**) Proportion of Lyz1 only positive cells (blue) and Venus and Lyz1 double positive (green) compared to total number of Lyz1 positive cells. (**c**) Proportion of Venus only positive cells (yellow) and double positive to Venus and Lyz1 (green) compared to total number of Venus cells. (**d**–**f**) Guca2a-Venus mice sections immunostained for Venus (GFP, green), lysozyme (red) and DNA (Hoechst; blue) from duodenum (**d**), jejunum (**e**) and ileum (**f**).

We further examined the cellular identity of *Guca2a* expressing cells by double immunostaining of tissue slices from *Guca2a*-Venus mice and by RT-qPCR of FACS-purified fluorescent cells. To address the overlap with Paneth cells, we examined expression of *Lyz1*, and immunostaining for lysozyme (Fig. 5). Confirming the RNAseq data, *Lyz1* was enriched in *Guca2a*-fluorescent cells from different regions of the small intestine (Fig. 5a). Dual staining for lysozyme and Venus (using an anti-GFP antibody) showed double labelled cells at the crypt bases in the duodenum, jejunum and ileum (Fig. 5d–f), with the majority of lysozyme positive cells being GFP positive: 67% in the duodenum, 91% in the jejunum and 86% in the ileum (Fig. 5b). Paneth cells represented ~50% of all Venus positive cells in the duodenum, but less than 15% in the jejunum and ileum, reflecting the higher overall frequencies of *Guca2a* positive cells in the jejunum and ileum (Fig. 5c).

**Figure 6:**
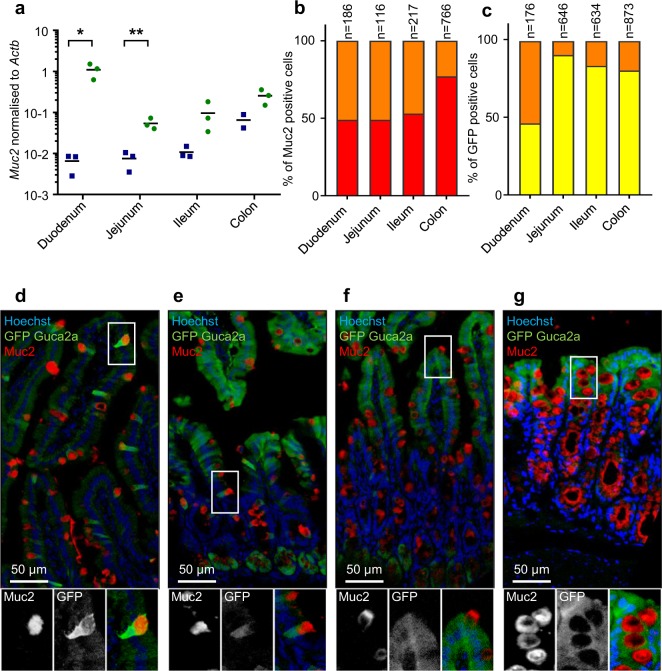
Guanylin expression in goblet cells. (**a**) Venus positive (green) and negative (blue) subpopulations were analysed by RT-qPCR for *Muc2* expression. n = 2–3 populations; positive and negative populations were compared using a paired t-test *p < 0.05 **p < 0.01. (**b**) Proportion of Muc2 only positive cells (red) and Venus and Muc2 double positive (orange) cells in comparison to the total number of Muc2 positive cells. (**c**) Proportion of Venus only positive cells (yellow) and Venus and Muc2 double positive (orange) cells in comparison to the total number of Venus positive cells. (**d**–**g**) Guca2a-Venus mice sections stained for Venus (GFP, green), muc2 (red) and DNA (Hoechst; blue) of duodenum (**d**), jejunum (**e**), ileum (**f**) and colon (**g**).

To validate and quantify the localisation of *Guca2a* in goblet cells, we measured expression of the markers *Muc2* by qPCR, and performed double staining for Muc2 and GFP in *Guca2a*-Venus tissues. Expression of *Muc2* was enriched in Venus-positive compared with negative cells (Fig. 6a), with the most marked differential expression evident in the duodenum. Many cells exhibited double-positive staining for Muc2 and GFP in all four tissues (Fig. 6c–g), with ~50% of all goblet cells staining positive for GFP in the duodenum, jejunum and ileum compared with ~20% in the colon (Fig. 6b). In the duodenum, 53% of all GFP positive cells exhibited co-staining for Muc2, whereas Muc2-positive goblet cells represented only 9%, 16% and 19% of the total GFP-positive cell populations in jejunum, ileum and colon, respectively, again reflecting the higher overall frequencies of fluorescent cells in these tissues (Fig. 6c).

**Figure 7:**
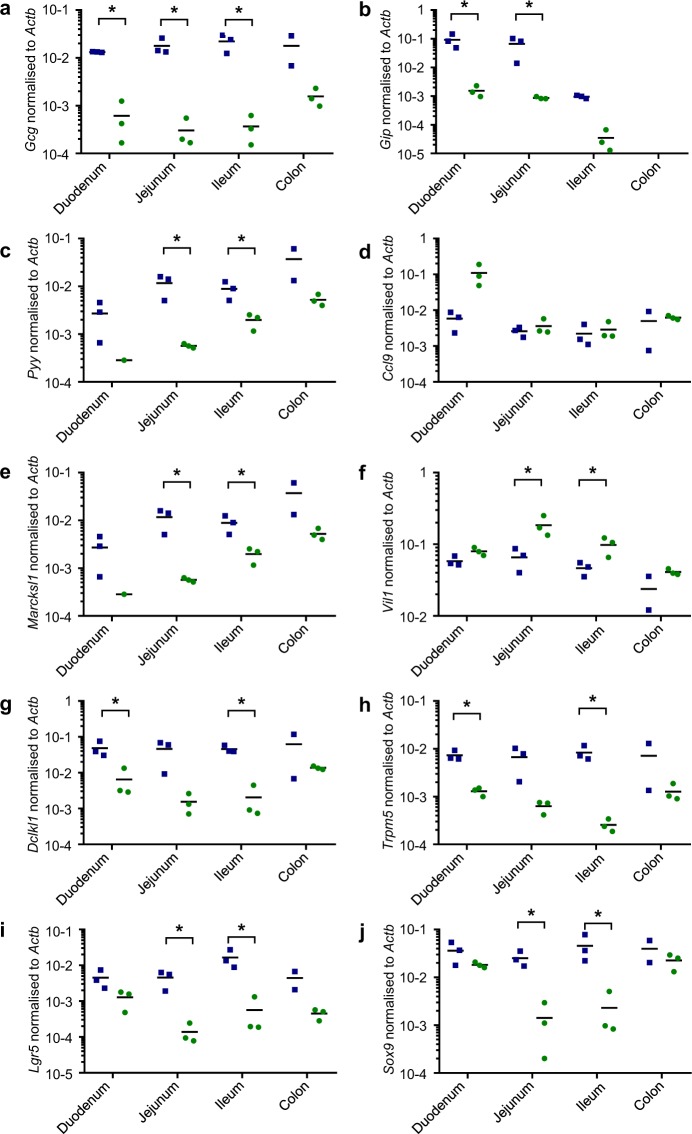
Other cell type markers. Gene expression levels of cell markers measured by RT-qPCR on cell-sorted Venus-positive and negative control subpopulations of Guca2a-Venus mice (n = 2–3). Markers for enteroendocrine cells: Gcg (**a**), Gip (**b**), Pyy(**c**), M cells: Ccl9 (**d**), Marcksl1 (**e**), enterocytes cells: Vil1 (**f**), tuft cells: Dclk1 (**g**), Trpm5 (**h**), Sox9 (**i**) and stem cells: Lgr5 (**j**) were checked for both negative (blue) and positive (green) subpopulation. Data are individual gene expression normalised to *β-Actin* and mean value is represented. Positive and negative populations were compared using a paired t-test. *p < 0.05.

Altogether, these results show that the *Guca2a* positive cell population in the duodenum is largely made up of Paneth and goblet cells, each representing about half of all *Guca2a* cells in this region. In the jejunum and ileum, Paneth and goblet cells together only explain a third of the *Guca2a* positive population, the remainder being mature enterocytes, as further supported by the expression of the different intestinal cell-type markers by qPCR (Fig. 7). In the colon, only ~20% of *Guca2a* positive cells stained positive for Muc2, and the remainder appeared to be dominated by mature colonocytes.

### Regulation of guanylin secretion

**Figure 8:**
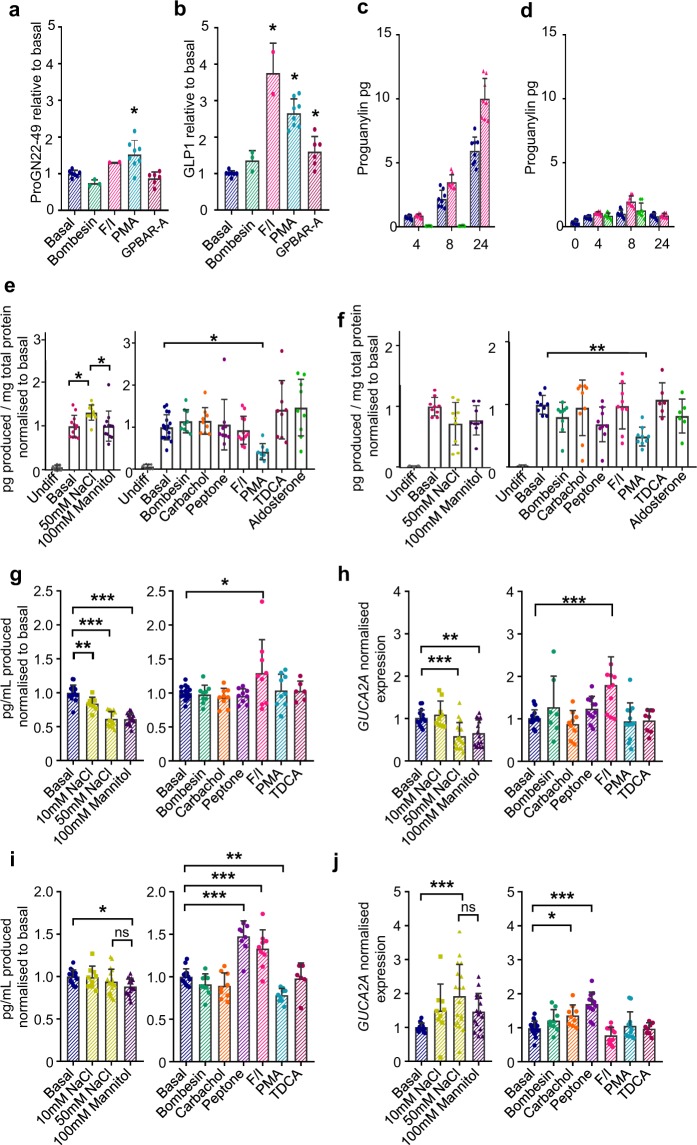
Proguanylin secretion *in vitro*. (**a**,**b**) Effect of different stimuli on proguanylin(22–49) (**a**) and GLP-1 (**b**) secretion from primary human colonic cultures, measured by LC-MS/MS after 1 h incubation in the absence (basal) or presence of bombesin (100 nM), forskolin/IBMX (10 μM each), PMA (1 μM) or GPBAR-A (3 μM). (**c**,**d**) Quantities of proguanylin in TC7 cell supernatants (**c**) and lysates (**d**) at different time points in standard media (blue) with forskolin/IBMX (10 μM each, red) or brefeldinA (5μg/mL, green). Data are mean ± SD, n = 8. (**e**,**f**) Effects of different stimuli on proguanylin secretion, measured by immunoassay, from human duodenal organoids (**e**) and human colonic organoids (**f**) Cultures were incubated with 50 mM NaCl, 100 mM mannitol, 100 nM bombesin, 1 mM carbachol, 5 mg/mL peptone, forskolin/IBMX (10 μM each), 1 μM PMA, 100 μM taurodeoxycholic acid (TDCA) or 100 nM aldosterone for 24 h. Data were normalised to total protein content and to the mean basal value. Data are mean ± SD, n = 6–18. (**g**–**j**) Secretion and expression of proguanylin in TC7 cells in response to different stimuli. TC7 cultures were incubated with stimuli as in (**e**). Supernatant proguanylin levels, measured by immunoassay (**g**,**i**) as well as transcript levels (**h**,**j**) were measured after 4 h incubation (**g**,**h**) and 24 h incubation. (**i**,**j**) Measured proguanylin concentrations were normalised to the mean basal value in control wells per experiment. Gene expression was calculated using the 2^ΔΔCt^ method, normalising to *β-act* and the relative expression in control (basal) wells for each experiment. Data are mean ± SD, n = 9–17 from 3–6 independent experiments. Responses were analysed by ANOVA followed by a Dunnett test. ***P < 0.001, **P < 0.01, *P < 0.05.

We next investigated whether proguanylin release could be modulated by a panel of candidate stimuli known to stimulate different epithelial cell types. In particular, we tested agents that could potentially target a variety of cell types, including PMA to activate PKC and forskolin/IBMX to increase cAMP concentrations; stimuli known to enhance secretion from enteroendocrine cells via cell surface receptors, including bile acids, peptones and bombesin; and stimuli previously reported to modulate guanylin release, including elevated salt concentrations and carbachol. Secretion was first measured from primary human colonic crypt cultures containing a variety of cell types, using LC-MS/MS to provide parallel quantification of a readily detectable proguanylin fragment 22–49 and GLP-1. After 1 h of incubation, proguanylin secretion was slightly enhanced by the PKC activator PMA, but was not altered by bombesin, forskolin/IBMX or an agonist of the Gs-protein coupled bile acid receptor GPBAR1, which robustly elevated GLP-1 (Fig. 8a,b).

We next used differentiated human duodenal and colonic organoids as a model of mixed epithelial cell types, to measure proguanylin secretion by immunoassay over 24 h in response to a panel of stimuli including sodium chloride, nutrients, bile acids and neurotransmitters. As expected, undifferentiated organoids had very low levels of GUCA2A expression and did not secrete detectable concentrations of proguanylin, whereas *GUCA2A* expression and proguanylin secretion increased following organoid differentiation (Fig. 8e,f and Supplementary Fig. 2). Differentiated organoids secreted proguanylin into the medium, and in the duodenum (but not colon) this was marginally stimulated by 50 mM NaCl but not 100 mM mannitol (Fig. 8e,f). No other condition modulated proguanylin secretion in either duodenal and colonic organoids, except for an inhibition seen with PMA, contrasting with the slight increase observed in primary cultures after more acute (1 h) stimulation.

We then assessed candidate mechanisms regulating proguanylin secretion using a human enterocyte cell line model, TC7, a subclone of the Caco-2 cell line. This more homogenous culture system also allowed us to monitor changes in the expression of proguanylin, either after acute (4 h) or more prolonged (24 h) stimulation. Addition of 10 or 50 mM NaCl, or 100 mM mannitol as an osmolarity control, resulted in a reduction in proguanylin secretion into the media after 4 hours, with the higher NaCl and mannitol treated cells also exhibiting a correspondingly lower expression of *GUCA2A* relative to the housekeeper gene *ACTB* (Fig. 8g,h). After 24 h incubation, however, proguanylin levels in salt and mannitol-treated wells were similar to those in control wells, and *GUCA2A* expression was elevated (Fig. 8i,j). We also measured proguanylin release and biosynthesis in TC7 cells in response to known enteroendocrine stimuli: bombesin, carbachol, peptones, taurodeoxycholic acid, PMA and forskolin/IBMX. After 4 h incubation, the only stimulus that significantly increased the release of proguanylin was 10 μM forskolin/IBMX, corresponding with a parallel increase in *GUCA2A* expression (Fig. 8g,h). After 24 h, proguanylin release and transcript levels were also increased by peptones, and a small reduction in secretion was found in PMA-treated wells (Fig. 8i,j). At this later timepoint with forskolin/IBMX, the *GUCA2A* transcript levels were not significantly different from controls (Fig. 8j).

As we had inconsistent results in these different culture systems, with only forskolin/IBMX apparently increasing proguanylin release in TC7 cells, we quantified proguanylin in cell supernatants and cell extracts at different time points over 24 hours. After 4 h, the amount of proguanylin in the supernatant, measured by immunoassay, was similar to that found in cell lysates, and after longer incubations the supernatant concentrations increased approximately linearly, whereas the lysate content stayed relatively constant (Fig. 8c,d). These results suggest that the cells turn over their entire proguanylin content in approximately 4 hours. In cells treated with forskolin/IBMX, slightly higher supernatant proguanylin concentrations were detected after 8 and 24 hours, corresponding with a small increase in the lysate content at 8 h. The slightly higher secretory rate in forskolin/IBMX might therefore reflect a stimulation by cAMP of proguanylin biosynthesis. When TC7 cells were treated for 4 or 8 hours with brefeldin A, an inhibitor of ER/Golgi transport, proguanylin secretion was largely abolished, although lysate concentrations remained unchanged (Fig. 8c,d), suggesting that ER/Golgi transport is required for proguanylin secretion, and that there is very little storage of proguanylin in post-Golgi compartments.

## Discussion

In this study, we applied new tools to investigate the cellular origin and secretion of guanylin peptides, including a new immunoassay for human proguanylin, LC-MS/MS analysis of proguanylin-derived peptides, and a transgenic mouse model expressing Venus under the control of the proguanylin promoter that enabled the identification, isolation and transcriptomic analysis of proguanylin-expressing cells. We found that proguanylin is constitutively secreted into the gut lumen, predominantly by mature enterocytes in the distal small and the large intestine, but in the duodenum only by Goblet and Paneth cells.

Guanylin and uroguanylin have been reported to exhibit differential expression along the GI tract, with *Guca2b* mainly expressed in the proximal small intestine and *Guca2a* mainly expressed in the distal small intestine and the colon^16,36^. Using the Guanylin-Venus transgenic mouse, we observed guanylin-dependent fluorescence in a variety of intestinal cell types and found that the purified fluorescent cell populations were enriched for both *Guca2a* and *Guca2b*. The pattern of Venus fluorescence in the duodenum was atypical compared with the rest of the GI tract, as it was largely restricted to Paneth and goblet cells. In the remainder of the gut, these cell types were also fluorescent, but we additionally observed expression in “mature” enterocytes at the small intestinal villus tips and the colonic surface epithelium. These results are in agreement with a recent RNAscope analysis^16^ which found a similar distribution of guanylin and uroguanylin along the murine rostro-caudal and crypt-villus intestinal axes. Unlike this murine RNA-scope analysis, we did not find tuft cell markers in guanylin-Venus cells, but our results are consistent with a recent study that failed to detect *GUCA2A* in human and rat tuft cells^37^, and another report suggesting that uroguanylin but not guanylin is expressed in this cell type^18^. In agreement with the RNA-scope studies^16,37^, we found no evidence for guanylin expression in enteroendocrine cells, contrasting with reports showing expression in human or rat enteroendocrine cells by immunohistochemistry^12,13,28^. Whilst these previous studies themselves are inconsistent by identifying different enteroendocrine subpopulations (EC-, D- and L-cells) to express guanylin, possibly due to antibody cross-reactivity, we cannot exclude possible species differences.

LC-MS/MS analysis of human plasma readily detected full length proguanylin, consistent with our measurement of high circulating proguanylin concentrations by immunoassay. Shorter proguanylin fragments were not detectable by LC-MS/MS in human plasma. In media from murine Ussing chamber studies, we detected full length proguanylin together with lower levels of a variety of cleaved forms. Our finding that full length proguanylin (22–116/115) is the major released form is consistent with previous reports in humans and rats^38–40^. Whilst we did not detect the originally described canonical sequence for the 15aa long active guanylin (mouse 102–116/human 101–115)^41^, we regularly and consistently detected peptides that were one amino acid shorter or longer at the N-terminus, as also reported previously for rats and humans^38,40^ thus suggesting that the originally isolated guanylin 15-mer likely represents an artefact of the acetic extraction method as previously proposed^42^. In murine tissue extracts, LC-MS/MS confirmed the previously-observed proximal to distal gradient of proguanylin-peptides, and identified a variety of proguanylin cleavage products, some of which matched those found to be secreted in the Ussing chamber studies (mouse 101–116 and 103–116). As we were not able to quantify the relative proportion of cleaved to full-length proguanylin in the tissue extracts, it is not clear how much proguanylin processing occurs intracellularly and how much depends on enzymatic cleavage in the gut lumen or bloodstream. Although the enzymes responsible for luminal proguanylin processing remain unknown, we speculate a role for meprins, which are zinc metalloproteinases found highly expressed in the small intestinal epithelial brush border^43^, with sequence specificity predicted to cleave proguanylin to release the 16 amino acid long guanylin peptide, but of course future research will be required to investigate if proguanylin is a substrate for these metalloendo-peptidases.

In agreement with previous studies on rat tissues^2,44^, we found that proguanylin is predominantly secreted into the gut lumen rather than at the basolateral side, in sharp contrast to enteroendocrine hormones such as GLP-1. This finding was observed in both mouse and human intestine and is consistent with the localisation of guanylin-expression in goblet and Paneth cells, which are specialised to secrete peptides and mucus in the luminal direction. Proguanylin was also, however, detectable in the basolateral compartment of Ussing chamber experiments, albeit at lower levels than in the luminal chamber, consistent with its detection in human plasma. As it seems unlikely that proguanylin arrives in the circulation following its diffusion/leakage out of the gut lumen across the epithelial layer, there should be an additional secretory pathway in the basolateral direction from a cell type that remains unclear. Our finding that proguanylin was undetectable in the plasma of a patient who temporarily lacked an intestine prior to transplantation, argues against the possibility that circulating proguanylin derives from a non-intestinal source. The observation that bowel transplant patients had raised fasting plasma proguanylin levels is interesting and in line with previous reports of elevated proguanylin expression after Roux-en-Y gastric bypass surgery^28^. The variety of operations performed, ranging from isolated small bowel transplants to multivisceral transplants including liver and pancreas, however, prevents currently a more detailed analysis of what might upregulate proguanylin secretion into the plasma after surgery.

We were unable to identify clear stimuli of guanylin secretion *in vitro*, although these studies were limited by the cellular models available. TC7 cells did not exhibit strong evidence of regulated proguanylin release, but cellular content had a rapid turnover and secretion was blocked by brefeldin A, arguing for a continuous constitutive vesicular secretory pathway. As TC7 cultures would not have contained goblet and Paneth cells, intestinal organoids and primary intestinal cultures were used as an alternative in the hope they might have contained a mixture of cell types, but these also failed to exhibit robust secretion in response to candidate stimuli. PMA inhibited proguanylin secretion from organoid cultures over a 24 hour incubation, whereas it had no effect on secretion from TC7 cells over a similar timeframe and slightly stimulated secretion from primary cultures during a 1 hour incubation. The underlying explanation for the different responses of the two models is unclear but might reflect the different incubation times and the presence of different cell types in organoids and primary cultures, whereas TC7 cells are of an enterocyte lineage. We were also unable to provide convincing evidence in support of previous reports that guanylin levels are linked to increased sodium levels^20^. Our data are best compatible with the idea that proguanylin secretion from enterocytes occurs constitutively, with limited storage of acutely recruitable vesicular pools, as proguanylin accumulated over time in TC7 supernatants, but not in the cells themselves. Previous reports of carbachol-triggered proguanylin release from intact tissue pieces mounted in Ussing chambers^2^ and bethanechol-stimulated release from perfused colonic preparations^44^ might reflect degranulation of goblet cells, which are known to respond to cholinergic stimulation^45^. Moro *et al*. also showed that bombesin-stimulated proguanylin release was inhibited by tetrodotoxin, suggesting the involvement of the enteric nervous system (ENS), possibly explaining the lack of a response seen in the here employed culture systems, as these would lack the ENS.

Proguanylin concentrations in human plasma averaged ~15 ng/ml, approximately 1000-fold higher than fasting plasma levels of gut hormones such as GLP-1, and were largely unaffected by food or glucose ingestion. Taken together with the finding that secretion *in vitro* was largely unresponsive to a range of candidate stimuli, our data do not support the idea that plasma proguanylin acts as a classical hormonal signal related to nutrient intake. Indeed, although elevated proguanylin levels were reported previously in bariatric patients, consistent with our own findings, administration of proguanylin or guanylin in mouse models did not influence food intake or glucose homeostasis^28^. As GC-C is predominantly expressed in the GI tract, it remains uncertain whether circulating proguanylin has any signalling role.

Proguanylin released into the intestinal lumen is cleaved into smaller peptides, and although we did not detect the classical 15 amino acid guanylin peptide, we consistently found slightly longer and shorter peptides that are likely to exhibit activity on the GC-C receptor. It has been hypothesised previously that guanylin secreted by Paneth cells deep inside the crypts might activate CFTR in neighbouring enterocytes thus sustaining luminal fluid flow from the crypt to villus, whilst guanylin secreted by goblet cells could similarly provide water for full mucin hydration^16^. Guanylin released from the villus tips could potentially target nearby or distal enterocytes, providing a paracrine/autocrine feedback mechanism to ensure adequate epithelial hydration^16^. As full length proguanylin has limited activity at the GC-C receptor^46^, enzymatic processing in the brush border is essential for activity.

Future studies will be required to identify specific release pathways involved in secretion from the different cell types producing proguanylin, the mechanisms and enzymes regulating active guanylin generation in the gut lumen, and the physiological roles (if any) of circulating proguanylin.

## Materials and Methods

Unless otherwise stated, all chemicals were supplied by Sigma-Aldrich.

### Ethical approvals

Ethical approval for collection of human plasma samples was granted by the following National Research Ethics Committees: East of England – Cambridgeshire and Hertfordshire (ref: 13/EE/0195), East of England – Norfolk (ref: 14/EE/1247); and East of England – Cambridge South (ref: 16/EE/0338). Ethical approval for use of human tissue pieces for cell culture, organoid generation and Ussing chamber studies was granted by the East of England – Cambridge Central Research Ethics Committee (ref: 09/H0308/24); surgical specimens were obtained under this ethical approval from Tissue Bank at Addenbrooke’s Hospital (Cambridge, UK). All human studies were conducted in accordance with the ethical standards of the Helsinki Declaration of 1975. All human participants gave written informed consent, either themselves or through their parent and/or legal guardian if they were under the age of 18 years old, and all methods were performed in accordance with the relevant guidelines and regulations.

Animal procedures during the generation of antibodies were approved by the Babraham Institute Animal Welfare and Ethical Review Body. Animal procedures involving the generation and/or use of Guanylin-Venus mice were approved by the University of Cambridge Animal Welfare and Ethical Review Body. All animal experiments were performed in accordance with the relevant guidelines and regulations under the UK Home Office project licences 70/8084, 70/7824 and PE5OF6065 and were carried out in accordance with the Animals (Scientific Procedures) Act 1986 Amendment Regulations (SI 2012/3039).

### Human blood samples

Healthy volunteers and patients who had previously undergone prophylactic total gastrectomy were recruited for oral glucose tolerance tests (50 or 75 g OGTTs) or mixed liquid meal (Ensure plus) and blood was collected just before and 60 minutes after ingestion into lithium-heparin coated tubes and centrifuged 10 min at 4 °C at 3500 g, plasma was stored at −80 °C until analysis^47^. Other blood samples were obtained from patients having received small bowel transplants and healthy volunteers after an overnight fast and serum samples were stored at −80 °C until analysis.

### Transgenic mice generation

BAC constructs containing the sequence of the yellow fluorescent protein *Venus* driven by the mouse guanylin promoter were made by the RedEt technique^48^. Briefly the sequence in the 3 exons coding for *Guca2a* (*Guanylin*) from the start codon to the stop codon in the murine-based BAC RP23-104G3 (Source Bioscience) was initially replaced by a counter-selection cassette RpsL-neo (GeneBridges) and subsequently by the Venus sequence^49^ using Red/ET recombination technology (Genebridges). The RpsL/Neo (FSD001/FSD002) or Venus (FSD003/FSD004) sequences were amplified by PCR with Phusion-polymerase adding *Guca2a*-gene specific 3′ and 5′ sequences (Table 1). Positive recombinants were isolated using appropriate antibiotic selection and characterised by PCR. The identity and correct positioning of the introduced Venus sequence was confirmed by direct sequencing using oligonucleotides FSD045-FSD076 (Table 1) and restriction analysis using the ZraI enzyme (NEB) for DNA fingerprinting. The BAC was purified using a Maxi-Prep (Qiagen) and diluted to 1 ng/μL into injection buffer (10 mM Tris-HCl (pH 7.5), 0.1 mM EDTA, 100 mM NaCl, 0.03 mM spermine, and 0.07 mM spermidine). The Central Biomedical Services (University of Cambridge) performed the pronuclear injection of ova derived from C57Bl6J/CBA F1 crosses, which were implanted into pseudopregnant females. Integration of the transgene was screened by PCR on the pups’ ear clips after extracting the DNA by proteinase K digestion using the primers GFPF1 and GFPR1 (Table 1). Experiments were performed on tissue derived from animals backcrossed into C57Bl6J to varying degree (3–8 generations), with no changes having been observed in the expression profile along the intestinal axis during the backcrossing procedure.

**Table 1:** Table of primers.

Primer	Purpose	Sequence (5′-3′)
FSD001	Replacement GN by rspL/neoR cassette forward	GCCCCACTGTTTACCCCAGGCACTAGTACTGGCCTGTTCTCTGCATTGCATACTGCTACCGGCCTGGTGATGATGGCGGGATCGT
FSD002	Replacement GN by rspL/neoR cassette reverse	CTTCTAGAAGATAGAGGGGCTTCCACATGGGCTGAGAGAAAGGCAAGCGATGTCACTCTAGAAGAACTCGTCAAGAAGGCGATAG
FSD003	Replacement GN by Venus cassette forward	GCCCCACTGTTTACCCCAGGCACTAGTACTGGCCTGTTCTCTGCATTGCATACTGCTACCATGGTGAGCAAGGGCGAGGAGCTGT
FSD004	Replacement GN by Venus cassette reverse	CTTCTAGAAGATAGAGGGGCTTCCACATGGGCTGAGAGAAAGGCAAGCGATGTCACTCTACTTGTACAGCTCGTCCATGCCGAGA
FSD045	To sequence GN-Venus + −5000bp f1	CTCCAGGAGGAGGAAAAAGAG
FSD046	To sequence GN-Venus + −5000bp f2	CAGTGAGCTCCAGCTCACTG
FSD047	To sequence GN-Venus + −5000bp f3	CAAACCGTGCCACTTTATTTC
FSD048	To sequence GN-Venus + −5000bp f4	CTGGCTTGCCAGAAGATGATG
FSD049	To sequence GN-Venus + −5000bp f5	CAGCAGCCTCCACACTCGAG
FSD050	To sequence GN-Venus + −5000bp f6	CTTTGCACACATGCCTTTCAG
FSD051	To sequence GN-Venus + −5000bp f7	CTTCTTCATCGAGGGCTTCC
FSD052	To sequence GN-Venus + −5000bp f8	CAGCAGATGAGGCTGACAAG
FSD053	To sequence GN-Venus + −5000bp f9	CTCCTGGGGTCACTAACTTGC
FSD054	To sequence GN-Venus + −5000bp f10	CCTGATCTTCCACATCCTGTG
FSD055	To sequence GN-Venus + −5000bp f11	GGTACACAGCTTCCTCCCAG
FSD056	To sequence GN-Venus + −5000bp f12	CTGAAAGCTGTCTCAGCATGG
FSD057	To sequence GN-Venus + −5000bp f13	GACAACCCAGGAAGCATCAAG
FSD058	To sequence GN-Venus + −5000bp f14	GAACACTTGGTCCCAGTAGGTG
FSD059	To sequence GN-Venus + −5000bp f15	GGCAGAGAGTAGCTGGGTGTAG
FSD060	To sequence GN-Venus + −5000bp f16	GGACGTGTCACTCCCTGATTG
FSD061	To sequence GN-Venus + −5000bp r1	GTGTTGCTTCTTTGATTGGTCTG
FSD062	To sequence GN-Venus + −5000bp r2	CAATCAGGGAGTGACACGTCC
FSD063	To sequence GN-Venus + −5000bp r3	CTACACCCAGCTACTCTCTGCC
FSD064	To sequence GN-Venus + −5000bp r4	CACCTACTGGGACCAAGTGTTC
FSD065	To sequence GN-Venus + −5000bp r5	CTTGATGCTTCCTGGGTTGTC
FSD066	To sequence GN-Venus + −5000bp r6	CCATGCTGAGACAGCTTTCAG
FSD067	To sequence GN-Venus + −5000bp r7	CTGGGAGGAAGCTGTGTACC
FSD068	To sequence GN-Venus + −5000bp r8	CACAGGATGTGGAAGATCAGG
FSD069	To sequence GN-Venus + −5000bp r9	GCAAGTTAGTGACCCCAGGAG
FSD070	To sequence GN-Venus + −5000bp r10	CTTGTCAGCCTCATCTGCTG
FSD071	To sequence GN-Venus + −5000bp r11	GGAAGCCCTCGATGAAGAAG
FSD072	To sequence GN-Venus + −5000bp r12	CTGAAAGGCATGTGTGCAAAG
FSD073	To sequence GN-Venus + −5000bp r13	CTCGAGTGTGGAGGCTGCTG
FSD074	To sequence GN-Venus + −5000bp r14	CATCATCTTCTGGCAAGCCAG
FSD075	To sequence GN-Venus + −5000bp r15	GAAATAAAGTGGCACGGTTTG
FSD076	To sequence GN-Venus + −5000bp r16	CAGTGAGCTGGAGCTCACTG
GFPF1	mouse screening	GACGTAAACGGCCACAAGTT
GFPR1	mouse screening	GGATCTTGAAGTTCGCCTTG

### Immunohistochemistry

Tissues from different regions of mouse gut were fixed in 4% PFA (Alfa Aesar) at room temperature overnight, dehydrated in sucrose (6 h 15% followed by 24 h 30%) at 4 °C. Tissue was embedded in OCT (VWR) and sectioned (7–10 μm) using a cryostat and mounted on Superfrost Plus glass slides (Thermo Fisher Scientific). Tissues sections were blocked in 10% donkey serum and 1% BSA and permeabilized with 0.05% Tween-20. Slides were stained with primary antibodies diluted in blocking solution over night at room temperature (RT) (Table 2). Slides were washed in PBS and 0.05% Tween-20 and then incubated with secondary antibodies conjugated to Alexa-Fluor 488, 555 or 633 (Invitrogen) and Hoechst nuclear stain (1:1300). Sections were mounted in hydromount (National Diagnostics) and images taken using a TCS SP8 confocal microscope using LAS software (Leica).

**Table 2:** Table of antibodies and antisera used.

Use	Antibody target	Company and catalog number	RRID	Dilution
hybridoma	FITC labelled monoclonal antibody anti-mouse IgG	Jackson ImmunoResearch (115-095-164)	AB_2338600	10 μg/mL
HTRF assay	AlexaFluor 647 labelled goat anti mouse Fc IgG	Jackson ImmunoResearch (115-605-164)	AB_2338913	7.5 nM
Cell sorting	anti-CD45-PE	Thermo Fisher (MA110233)	AB_11153376	1/500
IC	Donkey anti-mouse 647	Life technologies (A31571)	AB_162542	1/300
IC	Donkey anti-goat 488	Life technologies (A11055)	AB_2534102	1/300
IC	Donkey anti-rabbit 555	Life technologies (A31572)	AB_162543	1/300
IC	Goat anti-GFP	Abcam (Ab5450)	AB_304897	1/1000
IC	Rabbit anti-Lysozyme	Dako (A0099)	AB_2341230	1/200
IC	Rabbit anti-Muc2	Santa Cruz (sc15334)	AB_2146667	1/200
IC	Mouse anti-E-cadherin	BD (610181)	AB_397580	1/200

### Primary murine intestinal single-cell digestion for fluorescence-activated cell sorting

Four distinct parts of the gastrointestinal tract corresponding to the duodenum (top 3 cm of the small intestine), jejunum (3 cm in the middle small intestine) and ileum (bottom 3 cm of the small intestine) as well as the whole colon and rectum were treated separately and digested into single cells^50^. Each intestine part was opened longitudinally, rinsed in PBS and the outer muscle layer was removed. Each part was incubated in 10 mL solution containing 15 mM EDTA (or 30 mM for colon) and 1 mM DTT in Ca^2+^-free PBS for 7 minutes at RT. Tissue pieces were transferred into 15 mL Ca^2+^-free PBS with 10 μM ROCK inhibitor y27632 (Tocris) and shaken by hand for 30 seconds to collect epithelial fragments, and tissue transferred back to the EDTA solution. Incubation in EDTA and shaking was repeated 5 times. Epithelium fragments were kept on ice and after final shaking, centrifuged at 500 g at 4 °C and media was removed and replaced with 10 mL Trypsin 0.25% EDTA, 100 mg/mL DNAseI for 5 minutes at 37 °C. 15 mL of Hanks’ buffered salt solution (HBSS) supplemented with 10% FBS and 5 μM y27632 was added and cells triturated before centrifugation at 500 g at 4 °C for 5 minutes. Cells were resuspended in 10 mL washing solution (HBSS with 0.1% BSA and 5 μM y27632) and filtered through 100 µm and 50 µm cell filters. Single cells were pelleted again (500 g at 4 °C for 5 minutes), and resuspended in 150 µL of the washing solution containing an anti-CD45-PE antibody (1/500) (Thermo Fisher #MA110233) for 1 h on ice. Cells were centrifuged at 500 g at 4 °C for 5 minutes, resuspended in 200 µL washing solution containing DAPI and incubated for 5 minutes. Cells were washed twice with 1 mL washing solution and finally resuspended in 500 µL washing solution containing 5 µM DRAQ5 (Fisher Scientific). Single cell suspensions were sorted within 2 h on a FACSJazz (BD) at the Cambridge NIHR BRC Cell Phenotyping Hub. For each tissue, cells with high Venus fluorescence and non-fluorescent cells were sorted directly into lysis buffer (RLT plus, Qiagen) whilst dead cells, nucleus-free remnants and immune cells were excluded on the basis of DAPI, DRAQ5 and CD45 staining, respectively.

### RNA extraction, RNA sequencing and RT-qPCR

Total RNA from FACS-isolated cells was extracted using RNAeasy Micro Plus kit (Qiagen) according to the manufacturer’s instructions and concentration and quality assessed using a Bioanalyser RNA Pico kit (Agilent). RNA samples with RNA integrity number values between 8.6 and 10 were used. Libraries were prepared and amplified using SMARTer Pico V2 Mammalian prep kit (Takara) from 10 ng of RNA. Libraries were single-end 50 base pair sequenced at CRUK Cambridge on an Illumina HiSeq. 4000 platform. The average total number of reads in each sample was 32.3 million with an average of 21.4 million mapping uniquely to the mouse genome GRCm38 using STAR^51^. Read counts per gene were determined using STAR and differential gene expression was determined using DESeq. 2^52^. Raw data and generated count table are available with the GEO accession number GSE128117.

RNA from homogenised tissues and cell cultures were extracted using TRI Reagent following standard protocols. RNA was treated with DNAse I (Ambion), and reverse transcription was performed using the High-Capacity cDNA Reverse Transcription Kit (Applied Biosystems). RNA from sorted cells was reverse-transcribed using superscript III (Invitrogen) according to the manufacturer’s protocol. RT-qPCR was performed using the fast Taqman universal mastermix and the Taqman probes listed in Table 3 on a QuantStudio 7 (Applied Biosystems). Gene expression was measured relative to that of *β*-actin measured in parallel in the same sample using the ΔCt method.

**Table 3:** Table of Taqman assays probes.

Gene	Species	TaqMan™Gene Expression Assay Id
Guanylin (Guca2a)	mouse	Mm00433863_m1
Uroguanylin (Guca2b)	mouse	Mm01192051_m1
Beta actin (Actb)	mouse	Mm02619580_g1
Mucin 2 (Muc2)	mouse	Mm01276696_m1
Lysozyme (Lyz1)	mouse	Mm00657323_m1
SAM Pointed Domain Containing ETS Transcription Factor (Spdef gene)	mouse	Mm00600221_m1
SRY-Box 9 (Sox9)	mouse	Mm00448840_m1
Villin 1 (Vil1)	mouse	Mm00494146_m1
Glucagon (Gcg)	mouse	Mm01269055_m1
Gastric Inhibitory Polypeptide (Gip)	mouse	Mm00433601_m1
Peptide YY (Pyy)	mouse	Mm00520716_g1
Cholecystokinin (Cck)	mouse	Mm00446170_m1
Somatostatin (Sst)	mouse	Mm00436671_m1
Doublecortin Like Kinase 1 (Dclk1)	mouse	Mm00444950_m1
Transient Receptor Potential Cation Channel Subfamily M Member 5 (Trpm5)	mouse	Mm01129032_m1
Chemokine (C-C motif) ligand 9 (Ccl9)	mouse	Mm00441260_m1
Macrophage Myristoylated Alanine-Rich C Kinase Substrate (Marcksl1)	mouse	Mm00456784_m11
Leucine Rich Repeat Containing G Protein-Coupled Receptor 5 (Lgr5)	mouse	Mm00438890_m1
Guanylin (GUCA2A)	Human	Hs00157859_m1
Uroguanylin (GUCA2B)	Human	Hs00951189_m1
Beta actin (ACTB)	Human	Hs01060665_g1
Venus (Yfp)	Other	Mr03987581_mr

### Mass spectrometry

Plasma samples were extracted using ACN precipitation further purified using an HLB Prime μElution solid‐phase extraction (SPE) plate (Waters, MA, USA)^53^, whilst the Ussing chamber samples were extracted using SPE only. Peptides extracts were analysed using a Thermo Fisher Ultimate 3000 LC system coupled to a Q Exactive Plus Orbitrap mass spectrometer (ThermoScientific, San Jose, USA) as previously described^54^. LC-MS/MS data were analysed using Peaks v8.5 to identify peptides sequences on the *Mus musculus* and *Homo sapiens* Swissprot databases (downloaded on 26/10/2017) and XCalibur 4.1 (ThermoFisher) to quantify peptides measuring signal peak areas for specific sets of m/z and retention time corresponding to the identified peptides. Quantification of the guanylin peptides along the mouse intestine was analysed on previously published LC-MS/MS data (ProteomeXchange PXD009788, 10.6019/PXD009788)^47^.

### Antibody generation

The production and purification of soluble proguanylin in *E*. *coli* was described by Lauber and collaborators^55^. This method was used to produce 25 mg of proguanylin for the hybridoma generation, screening and assay development. The purified protein was subjected to endotoxin removal using Polymyxin B resin^56^. To perform the primary screen of the hybridoma clones and IgG characterisation, 2 mg of proguanylin was enzymatically biotinylated using the BirA enzyme (Avidity LLC #BirA500) according to manufacturer’s instructions. Female CD1 mice aged 6–8 weeks were used for hybridoma generation and injected subcutaneously with 100 µg of recombinant proguanylin followed by 3 boosts of 100 µg each over 21 days. The immunisation, hybridoma generation and cDNA preparation and variable chain sequencing and IgG purification were performed as previously described^57^. For the clone selection, a Homogeneous Time Resolved Fluorescence biochemical assay was developed to measure the binding of IgGs to the biotinylated targets using Europium cryptate conjugated streptavidin (Cisbio #610SAKLB) as the donor and AlexaFluor 647 conjugated goat anti-mouse Fc IgG (Jackson #115-605-164) as the acceptor. Test samples were incubated for 15 h at RT with 2.5 nM biotinylated-Proguanylin premixed with 1 nM Streptavidin-cryptate, and 7.5 nM AlexaFluor 647 conjugated goat anti-mouse Fc IgG. The fluorescence was measured on an EnVision plate reader (Perkin Elmer) using a 320 nm excitation filter and 665 nm and 590 nm emission filters.

### Cell culture

TC7 cells (RRID:CVCL_0233, a sub-clone of the caco-2 cell line^58^) were previously characterized as differentiating epithelial cells^59^ and were cultured in Dulbecco’s modified Eagle’s medium (DMEM, 4.5 g/L glucose) supplemented with 10% Fetal Bovine Serum (FBS), 100 units/mL penicillin, 100 μg/mL streptomycin and 2 mM L-glutamine at 37 °C, 5% CO_2_ and passaged every 7 days.

Human organoid lines were generated using a modified protocol^60,61^. Briefly, fresh surgical specimens of fresh human duodenum and colon were minced, and fragmented with 30 mM EDTA for 3 × 10 mins, with tissue shaken in PBS after each EDTA treatment. The fraction with intestinal crypts was then further digested using TrypLE (Life Technologies) for 5 mins at 37 °C to generate small cell clusters, which were seeded into basement membrane extract (BME, R&D technology), with 20 μl domes polymerised in multiwell dishes for 30–60 mins at 37 °C. Organoid medium^61^ was then overlaid and changed 3 times per week. Organoids were passaged every 10–21 days, with TrypLE digestion for 15 mins at 37 °C followed by mechanical shearing with rigorous pipetting for 30 times to breakup organoids into small clusters which were then seeded as before in BME. To generate differentiated cell types, organoid domes were washed 3 times in Advanced DMEM/F12 over 1 h. Organoid media lacking wnt3A, SB202190, A83-01 and nicotinamide was then added to the wells and changed every day for up to 10 days.

### Secretion assay

Mouse primary cultures were prepared as described^62^. Briefly, colons from individual mice were harvested and digested with 0.4 mg/mL collagenase XI. Isolated crypts were plated on matrigel overnight and the day after, cells were rinsed with secretion buffer ((in mM) 5.6 KCl, 138 NaCl, 4.2 NaHCO_3_, 1.2 NaH_2_ PO_4_, 2.6 CaCl_2_, 1.2 MgCl_2_, and 10 HEPES, pH 7.4 and 0.001% BSA) 3 times over 30 minutes and incubated in secretion buffer with indicated drugs for 1 h. Supernatant was then collected and centrifuged 5 min at 2,000 g at 4 C and transferred to a fresh Protein Lobind tube before purification through an SPE plate and analysis by LC-MS/MS as described.

Differentiated TC7 cells (grown at confluency for 9 days) or 8–9 day old differentiated human organoids were used for secretion experiments. The wells were washed 3 times in culture medium. Test agents were dissolved in the same medium and 250 μL applied for TC7 secretions, or 150 μL applied for organoid secretions. TC7 cells or organoids were incubated at 37 °C for 4 h, 8 h, or 24 h. Medium was centrifuged for 10 mins at 350 g at 4 °C, with supernatant used for downstream analysis. For experiments that required cell lysates to measure total proforms or total protein levels, the cells were lysed in 150 μL of lysis buffer (50 mM Tris-HCl, 150 mM NaCl, 1% IGEPAL-CA630, 0.5% deoxycholic acid, and one tablet per 50 mL of complete EDTA-free protease inhibitor cocktail (Roche)) on ice for 30 mins, centrifuged at 10,000 g for 10 mins at 4 °C and the supernatants analysed.

Ussing chamber secretion experiments were performed with minor modifications as described previously^63^.Human and mouse intestinal tissue was transported to the laboratory in ice-cold L15 medium. Serosa and most of the muscular layers were stripped away with fine forceps and the remaining tissue was mounted in an Ussing chamber (EM-LVSYS-4 system with P2400 chambers and P2405 sliders; Physiologic Instruments). The active epithelial surface area of each segment was 0.4 cm^2^. Both parts of the Ussing chambers were filled with Ringer solution containing 120 mM NaCl, 3 mM KCl, 0.5 mM MgCl_2_, 1.25 mM CaCl_2_, and 23 mM NaHCO_3_, with 10 mmol/L glucose, maintained at 37 °C, and continuously bubbled with 5% CO_2_/95% O_2_ (vol/vol). The transepithelial potential difference was monitored under open circuit conditions, using a DVC 1000 amplifier (WPI) and recorded through Ag-AgCl electrodes and 150 mM NaCl agarose bridges. After 20–30 minutes, solutions from both sides were exchanged with 1.2 mL of fresh Ringer solution containing 10 mM glucose, 0.1% fatty acid–free BSA, 10 μM amastatin, 500 kIU/mL aprotinin and Infacol (Forest Laboratories, 1:10^5^ dilution, to prevent extensive foam formation). Solutions for LC-MS/MS only contained the additives 10 mM glucose and 0.001% BSA. Samples (120 μL) were taken from both compartments typically at 10, 40 and 85 minutes after solution exchange. IBMX (100 μM) and forskolin (10 μM) were added bilaterally immediately after the second sample was taken at 40 min. Reported secretion was normalized for 1 cm^2^ of the tissue and a 60-minute secretion period. GLP-1 and Proguanylin were measured by MSD immunoassay or LC-MS/MS as indicated.

### MSD assay

Anti-ProGuanylin antibody AB1000025 was coated using 5 µL of 50 ng/µL on an assay plate (MSD #MA6000) incubated at RT overnight. Plates were blocked using 1% BSA in PBS for 2 h at RT. After washing 3 times with washing buffer (PBS-Tween (0.05%)), 25 µL of samples were added to each well and incubated for 2 h at RT. Following incubation, plates were washed 3 times with washing buffer and 25 µL of a second Sulfo-Tag labelled anti-ProGuanylin antibody AB1000028 prepared at 1 µg/mL in Diluent 41 (MSD) was added per well for 1 h at RT. Once washed with washing buffer, 150 µL of Read buffer 2X was added to each well before reading the plate immediately using a MSD Workbench. A standard curve was constructed by fitting a sigmoidal function to the absorbance obtained from the known proguanylin concentrations and the concentration of experimental samples was assessed by interpolating them to the standard curve using the MSD discovery software. Samples in lysis buffer were diluted 1/20, dilution at which the lysis solution had no effect on measurements. GLP-1 concentrations in Ussing chamber experiments were measured at the Core Biochemical Assay Laboratory (Cambridge) using the total GLP-1 assay kit from MSD (K150JVC, RRID AB_2801383).

## Supplementary information

Supplementary Figures

## Data Availability

The datasets generated during and/or analyzed during the current study, the animal model (Guanylin-Venus) and the immuno-assay developed during this study are not publicly available but are available from the corresponding author on reasonable request. The RNAseq data has been deposited as outlined in material and methods.

## References

[1] Hamra FK (1993). Uroguanylin: structure and activity of a second endogenous peptide that stimulates intestinal guanylate cyclase. Proc. Natl. Acad. Sci. USA.

[2] Martin S, Adermann K, Forssmann W-G, Kuhn M (1999). Regulated, Side-Directed Secretion of Proguanylin from Isolated Rat Colonic Mucosa1. Endocrinology.

[3] Krause G, Bayerl A, Heim JM, Singh S, Gerzer R (1994). Distribution of membrane bound guanylyl cyclases in human intestine. Gut.

[4] Almenoff JS, Williams SI, Scheving LA, Judd AK, Schoolnik GK (1993). Ligand-based histochemical localization and capture of cells expressing heat-stable enterotoxin receptors. Mol. Microbiol..

[5] Nokihara K, Wray V, Ando E, Naruse S, Hayakawa T (1997). Synthesis, solution structure, binding activity, and cGMP activation of human guanylin and its disulfide isomer. Regul. Pept..

[6] Vaandrager AB (2002). Structure and function of the heat-stable enterotoxin receptor/guanylyl cyclase C. Mol. Cell. Biochem..

[7] Fiskerstrand T (2012). Familial Diarrhea Syndrome Caused by an Activating *GUCY2C* Mutation. N. Engl. J. Med..

[8] Müller, T. *et al*. Congenital secretory diarrhoea caused by activating germline mutations in GUCY2C. *Gut* **3**, gutjnl-2015-309441- (2015). 10.1136/gutjnl-2015-309441 PMC4975829 25994218

[9] Romi H (2012). Meconium ileus caused by mutations in GUCY2C, encoding the CFTR-activating guanylate cyclase 2C. Am. J. Hum. Genet..

[10] de Sauvage FJ (1992). Precursor structure, expression, and tissue distribution of human guanylin. Proc. Natl. Acad. Sci. USA.

[11] Cetin Y (1994). Enterochromaffin cells of the digestive system: cellular source of guanylin, a guanylate cyclase-activating peptide. Proc. Natl. Acad. Sci..

[12] Hill O (1995). Analysis of the human guanylin gene and the processing and cellular localization of the peptide. Proc. Natl. Acad. Sci..

[13] Ieda H (1998). Coexistence of proguanylin (1–15) and somatostatin in the gastrointestinal tract. J. Gastroenterol. Hepatol..

[14] Li Z, Taylor-Blake B, Light AR, Goy MF (1995). Guanylin, an endogenous ligand for C-type guanylate cyclase, is produced by goblet cells in the rat intestine. Gastroenterology.

[15] Rubio CA (2012). Paneth cells and goblet cells express the neuroendocrine peptide synaptophysin. I- normal duodenal mucosa. In Vivo (Brooklyn)..

[16] Ikpa PT (2016). Guanylin and uroguanylin are produced by mouse intestinal epithelial cells of columnar and secretory lineage. Histochem. Cell Biol..

[17] Whitaker TL, Witte DP, Scott MC, Cohen MB (1997). Uroguanylin and guanylin: distinct but overlapping patterns of messenger RNA expression in mouse intestine. Gastroenterology.

[18] Kokrashvili, Z. *et al*. Release of Endogenous Opioids From Duodenal Enteroendocrine Cells Requires Trpm5, 10.1053/j.gastro.2009.02.070 (2009). 10.1053/j.gastro.2009.02.070 PMC2717179 19272386

[19] Kinoshita H (1997). Urine and plasma levels of uroguanylin and its molecular forms in renal diseases. Kidney Int..

[20] Kita, T., Kitamura, K., Sakata, J. & Eto, T. Marked increase of guanylin secretion in response to salt loading in the rat small intestine. *Am*. *J*. *Physiol*. *- Gastrointest*. *Liver Physiol*. **277** (1999). 10.1152/ajpgi.1999.277.5.G960 10564101

[21] Carrithers S, Jackson B, Cai W, Greenberg R, Ott C (2002). Site-specific effects of dietary salt intake on guanylin and uroguanylin mRNA expression in rat intestine. Regul. Pept..

[22] Li P (2007). Homeostatic control of the crypt-villus axis by the bacterial enterotoxin receptor guanylyl cyclase C restricts the proliferating compartment in intestine. Am J Pathol.

[23] Wilson C (2014). The paracrine hormone for the GUCY2C tumor suppressor, guanylin, is universally lost in colorectal cancer. Cancer Epidemiol. Biomarkers Prev..

[24] Hoos A (2001). High Ki-67 proliferative index predicts disease specific survival in patients with high-risk soft tissue sarcomas. Cancer.

[25] Li LT, Jiang G, Chen Q, Zheng JN (2015). Ki67 is a promising molecular target in the diagnosis of cancer (Review). Mol. Med. Rep..

[26] Weinberg DS (2017). Bioactivity of Oral Linaclotide in Human Colorectum for Cancer Chemoprevention. Cancer Prev. Res. (Phila)..

[27] Scarpignato C, Blandizzi C (2014). Editorial: adequate management may reduce the colorectal cancer risk associated with constipation. Aliment. Pharmacol. Ther..

[28] Fernandez-Cachon ML (2018). Guanylin and uroguanylin mRNA expression is increased following Roux-en-Y gastric bypass, but guanylins do not play a significant role in body weight regulation and glycemic control. Peptides.

[29] Lin JE (2016). Obesity-induced colorectal cancer is driven by caloric silencing of the guanylin-GUCY2C paracrine signaling axis. Cancer Res..

[30] Valentino MA (2011). A uroguanylin-GUCY2C endocrine axis regulates feeding in mice. J. Clin. Invest..

[31] Folgueira, C. *et al*. Uroguanylin action in the brain reduces weight gain in obese mice via different efferent autonomic pathways. *Diabetes*, 10.2337/db15-0889 (2016). 10.2337/db15-0889 26566631

[32] Friedlander RS (2011). Role of phosphodiesterase and adenylate cyclase isozymes in murine colonic glucagon-like peptide 1 secreting cells. Br. J. Pharmacol..

[33] Waldman SA, Camilleri M (2018). Guanylate cyclase-C as a therapeutic target in gastrointestinal disorders. Gut.

[34] Roberts, G. P. *et al*. Comparison of human and murine enteroendocrine cells by transcriptomic and peptidomic profiling. *bioRxiv* 374579, 10.1101/374579 (2018). 10.2337/db18-0883 PMC6477899 30733330

[35] Rothenberg ME (2012). Identification of a cKit(+) colonic crypt base secretory cell that supports Lgr5(+) stem cells in mice. Gastroenterology.

[36] Qian X, Prabhakar S, Nandi A, Visweswariah SS, Goy MF (2000). Expression of GC-C, a Receptor-Guanylate Cyclase, and Its Endogenous Ligands Uroguanylin and Guanylin along the Rostrocaudal Axis of the Intestine ^1^. Endocrinology.

[37] Brenna Ø (2016). Cellular localization of guanylin and uroguanylin mRNAs in human and rat duodenal and colonic mucosa. Cell Tissue Res..

[38] Nakazato M (1994). Identification of 10-kDa Proguanylin as a Major Guanylin Molecule in Human Intestine and Plasma and Its Increase in Renal Insufficiency. Biochem. Biophys. Res. Commun..

[39] Kuhn M (1993). The circulating bioactive form of human guanylin is a high molecular weight peptide (10.3 kDa). FEBS Lett..

[40] Yamaguchi H (1995). Two Novel Rat Guanylin Molecules, Guanylin-94 and Guanylin-16, Do Not Increase Cyclic GMP Production in T84 Cells. Biochem. Biophys. Res. Commun..

[41] Currie MG (1992). Guanylin: an endogenous activator of intestinal guanylate cyclase. Proc. Natl. Acad. Sci. USA.

[42] Schulz S, Chrisman TD, Garbers DL (1992). Cloning and expression of guanylin. Its existence in various mammalian tissues. J. Biol. Chem..

[43] Sterchi EE, Naim HY, Lentze MJ, Hauri H-P, Fransen JAM (1988). N-benzoyl-l-tyrosyl-p-aminobenzoic acid hydrolase: A metalloendopeptidase of the human intestinal microvillus membrane which degrades biologically active peptides. Arch. Biochem. Biophys..

[44] Moro F (2000). Release of Guanylin Immunoreactivity from the Isolated Vascularly Perfused Rat Colon*. Endocrinology.

[45] Halm DR, Halm ST (2000). Secretagogue response of goblet cells and columnar cells in human colonic crypts ^1^. Am. J. Physiol. Physiol..

[46] Schulz A (1999). Role of the prosequence of guanylin. Protein Sci..

[47] Roberts, G. P. *et al*. Comparison of Human and Murine Enteroendocrine Cells by Transcriptomic and Peptidomic Profiling. *Diabetes* db180883, 10.2337/db18-0883 (2019). 10.2337/db18-0883 PMC6477899 30733330

[48] Zhang Y, Muyrers JPP, Testa G, Stewart AF (2000). DNA cloning by homologous recombination in Escherichia coli. Nat. Biotechnol..

[49] Rekas A, Alattia J-R, Nagai T, Miyawaki A, Ikura M (2002). Crystal Structure of Venus, a Yellow Fluorescent Protein with Improved Maturation and Reduced Environmental Sensitivity* Downloaded from. J. Biol. Chem..

[50] Larraufie P (2019). Important Role of the GLP-1 Axis for Glucose Homeostasis after Bariatric Surgery. Cell Rep..

[51] Dobin A (2013). STAR: ultrafast universal RNA-seq aligner. Bioinformatics.

[52] Love MI, Huber W, Anders S (2014). Moderated estimation of fold change and dispersion for RNA-seq data with DESeq. 2. Genome Biol..

[53] Kay RG (2018). Peptidomic analysis of endogenous plasma peptides from patients with pancreatic neuroendocrine tumours. Rapid Commun. Mass Spectrom..

[54] Kay, R. G., Galvin, S., Larraufie, P., Reimann, F. & Gribble, F. M. LC/MS based detection and semi-quantitative analysis of INSL5 in human and murine tissues. *Rapid Commun*. *Mass Spectrom*. **31** (2017). 10.1002/rcm.7978 PMC5698736 28857318

[55] Lauber T, Neudecker P, Rösch P, Marx UC (2003). Solution structure of human proguanylin: The role of a hormone prosequence. J. Biol. Chem..

[56] Vesentini, S., Soncini, M., Fiore, G. B. & Redaelli, A. *Mechanisms of Polymyxin B Endotoxin Removal from Extracorporeal Blood Flow: Molecular Interactions Sepsis is a generalized infection of an organism with presence of bacteria in the blood flow (bacteremia)*. *Recently*, *in parallel to clinical and research*. *Therapy*. *Contrib Nephrol*. *Basel*, *Karger* **167** (2010). 10.1159/000315918 20519898

[57] Percival-Alwyn JL (2015). Generation of potent mouse monoclonal antibodies to self-proteins using T-cell epitope ‘tags’. MAbs.

[58] Caro I (1995). Characterisation of a newly isolated Caco-2 clone (TC-7), as a model of transport processes and biotransformation of drugs. Int. J. Pharm..

[59] Goldspink, D. A. *et al*. Ninein is essential for apico-basal microtubule formation and CLIP-170 facilitates its redeployment to non-centrosomal microtubule organizing centres. *Open Biol*. **7** (2017). 10.1098/rsob.160274 PMC5356440 28179500

[60] Goldspink, D. A. *et al*. Mechanistic insights into the detection of free fatty and bile acids by ileal glucagon-like peptide-1 secreting cells. *Mol*. *Metab*. **7** (2018). 10.1016/j.molmet.2017.11.005 PMC5784317 29167062

[61] Sato T (2011). Long-term Expansion of Epithelial Organoids From Human Colon, Adenoma, Adenocarcinoma, and Barrett’s Epithelium. Gastroenterology.

[62] Psichas, A., Tolhurst, G., Brighton, C. A., Gribble, F. M. & Reimann, F. Mixed Primary Cultures of Murine Small Intestine Intended for the Study of Gut Hormone Secretion and Live Cell Imaging of Enteroendocrine Cells. *J*. *Vis*. *Exp*., 10.3791/55687 (2017). 10.3791/55687 PMC5409300 28448057

[63] Brighton CA (2015). Bile Acids Trigger GLP-1 Release Predominantly by Accessing Basolaterally Located G Protein-Coupled Bile Acid Receptors. Endocrinology.

