# Supplementary material for: Characterisation of proguanylin expressing cells in the intestine – evidence for constitutive luminal secretion
Source: Sci Rep. 2019 Oct 30;9:15574. doi: 10.1038/s41598-019-52049-0 (PMC6821700; doi:10.1038/s41598-019-52049-0)
Supplement: Supplementary file 1 — Supplementary Figures [file 41598_2019_52049_MOESM1_ESM.pdf]

Supplementary material for:

Dye FS, Larraufie P, Kay R, Darwish T, Rievaj J, Goldspink DA, Meek CL, Middleton SJ, Hardwick RH , Roberts GP, Percival-Alwyn JL, Vaughan T, Ferraro F, Challis BG, O'Rahilly S, Groves M, Gribble FM, Reimann F:

"Characterisation of proguanylin expressing cells in the intestine – evidence for constitutive luminal secretion"

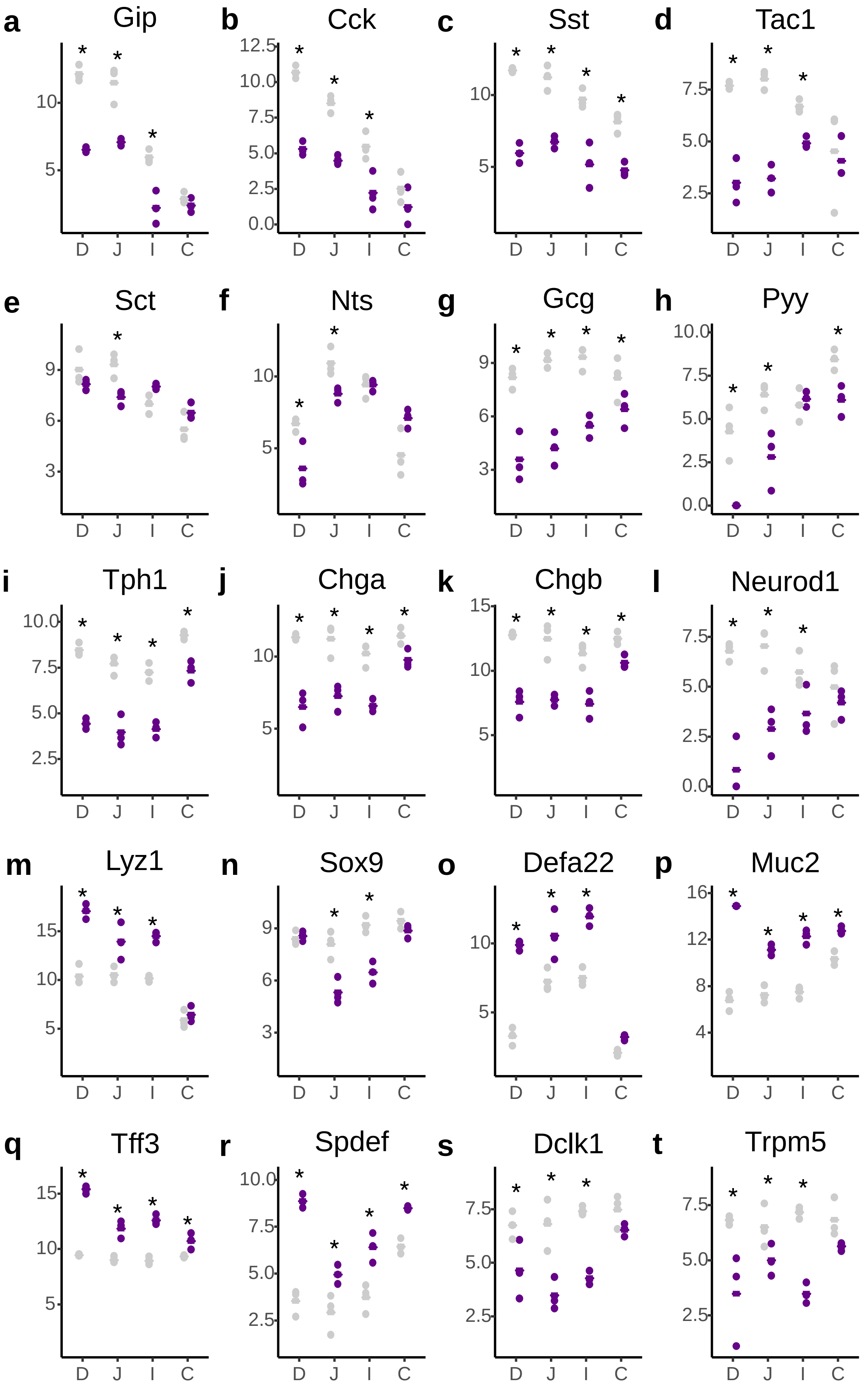

a Gip
10
5
D J I C
b Cck
12.5
10.0
7.5
5.0
2.5
0.0
D J I C
c Sst
10
5
D J I C
d Tac1
7.5
5.0
2.5
D J I C
e Sct
9
6
3
D J I C
f Nts
10
5
D J I C
g Gcg
9
6
3
D J I C
h Pyy
10.0
7.5
5.0
2.5
0.0
D J I C
i Tph1
10.0
7.5
5.0
2.5
D J I C
j Chga
10
5
D J I C
k Chgb
15
10
5
D J I C
l Neurod1
7.5
5.0
2.5
0.0
D J I C
m Lyz1
15
10
5
D J I C
n Sox9
9
6
3
D J I C
o Defa22
10
5
D J I C
p Muc2
16
12
8
4
D J I C
q Tff3
15
10
5
D J I C
r Spdef
10.0
7.5
5.0
2.5
D J I C
s Dclk1
7.5
5.0
2.5
D J I C
t Trpm5
7.5
5.0
2.5
D J I C

Supplementary Figure 1: Expression of secretory cell markers

Expression of secretory cell markers in Guca2a positive (purple) and negative (grey) cells across the different tissues (D: duodenum, J: jejunum, I: ileum, C: Colon). Data is shown as log-transformed Deseq2-normalised read counts for all samples and the mean for each group. * indicates that the gene is differently expressed between the positive and negative cells in the tissue (padj < 0.05) using Deseq2.

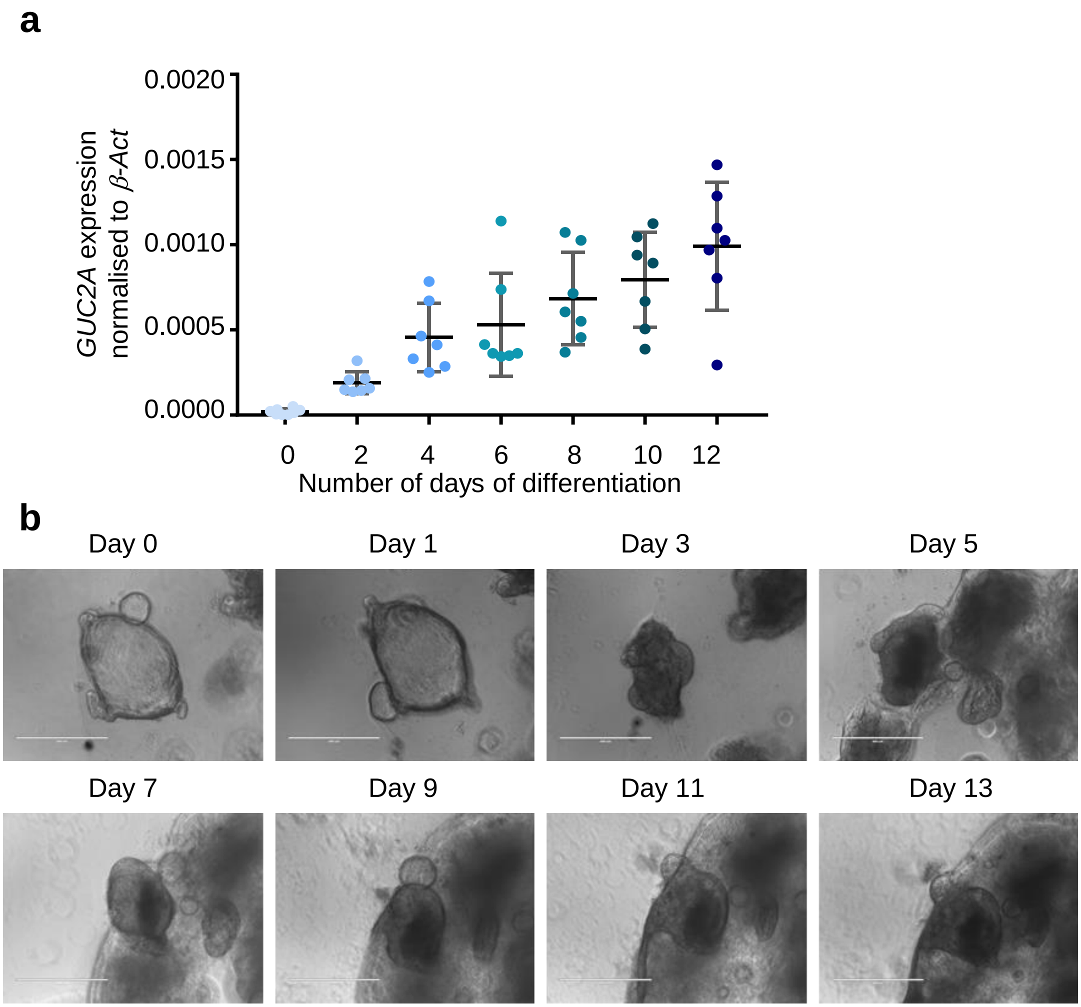

Supplementary Figure 2: Guanylin expression in differentiating organoids

(a) Expression of *GUCA2* measured by RT-qPCR in organoids after initiation of differentiation for the indicated number of days through incubation in differentiation media. Data are from 7 individual wells from n=3 independent experiments and mean ± sd. (b) Phase contrast images of an organoid monitored whilst incubated in differentiation media for 13 days (scale bar: 400 μm).
